# Impact of ghrelin on body composition and muscle function in a long-term rodent model of critical illness

**DOI:** 10.1371/journal.pone.0182659

**Published:** 2017-08-10

**Authors:** Neil E. Hill, Kevin G. Murphy, Saima Saeed, Rahul Phadke, Darren Chambers, Duncan R. Wilson, Stephen J. Brett, Mervyn Singer

**Affiliations:** 1 Section of Investigative Medicine, Imperial College London, London, United Kingdom; 2 Bloomsbury Institute of Intensive Care Medicine, Division of Medicine, University College London, London, United Kingdom; 3 Academic Department of Military Medicine, Royal Centre for Defence Medicine, Birmingham, United Kingdom; 4 Division of Neuropathology, UCL Institute of Neurology and National Hospital for Neurology and Neurosurgery, London, United Kingdom; 5 Dubowitz Neuromuscular Centre and MRC Centre for Neuromuscular Disorders, University College London Institute of Child Health and Great Ormond Street Hospital for Children, London, United Kingdom; 6 Centre for Perioperative Medicine and Critical Care Research, Imperial College Healthcare NHS Trust, London, United Kingdom; West Virginia University School of Medicine, UNITED STATES

## Abstract

**Background:**

Patients with multiple injuries or sepsis requiring intensive care treatment invariably develop a catabolic state with resultant loss of lean body mass, for which there are currently no effective treatments. Recovery can take months and mortality is high. We hypothesise that treatment with the orexigenic and anti-inflammatory gastric hormone, ghrelin may attenuate the loss of body mass following critical illness and improve recovery.

**Methods:**

Male Wistar rats received an intraperitoneal injection of the fungal cell wall derivative zymosan to induce a prolonged peritonitis and consequent critical illness. Commencing at 48h after zymosan, animals were randomised to receive a continuous infusion of ghrelin or vehicle control using a pre-implanted subcutaneous osmotic mini-pump, and continued for 10 days.

**Results:**

Zymosan peritonitis induced significant weight loss and reduced food intake with a nadir at Day 2 and gradual recovery thereafter. Supra-physiologic plasma ghrelin levels were achieved in the treated animals. Ghrelin-treated rats ate more food and gained more body mass than controls. Ghrelin increased adiposity and promoted carbohydrate over fat metabolism, but did not alter total body protein, muscle strength nor muscle morphology. Muscle mass and strength remained significantly reduced in all zymosan-treated animals, even at ten days post-insult.

**Conclusions:**

Continuous infusion of ghrelin increased body mass and food intake, but did not increase muscle mass nor improve muscle function, in a long-term critical illness recovery model. Further studies with pulsatile ghrelin delivery or additional anabolic stimuli may further clarify the utility of ghrelin in survivors of critical illness.

## Introduction

Loss of muscle mass following serious injury and critical illness is near inevitable. Muscle loss occurs as a result of atrophy occurring as a consequence of immobility and the disease process itself. Failure to recover adequately impacts significantly on later quality of life, including independent living and return to work [1].

Patients surviving the acute phase of their critical illness invariably become very cachetic, weak and immunosuppressed. The ability to recover functional muscle strength is obviously vital to short-term prognosis, for example weaning off mechanical ventilation and mobilizing, and is increasingly recognized as crucial to both long-term survival and survivorship [1,2]. Strategies to enhance recovery include early rehabilitation and mobilization [2] and both propranolol [3] and oxandralone [4] have been shown to enhance lean body mass in children with severe burns. Outside of the burns arena however, trials of hormone therapy have proved detrimental, as in the case of growth hormone [5] and thyroxine [6] while there is no clear protocol for the optimal amount, delivery and constituents of nutritional support [7].

The gastric hormone, ghrelin has both appetite-stimulating and immunomodulatory effects and offers a potentially useful therapy to enhance recovery from critical illness. We have previously reported significantly reduced plasma ghrelin levels in patients on day 1 of their intensive care admission that normalized over the following month [8]. Ghrelin was positively correlated with food intake, suggesting a relationship between critical illness, appetite and ghrelin levels. Notably, food intake remained significantly below estimated energy requirements at four weeks.

The therapeutic potential of ghrelin in critical illness has been explored in several laboratory studies [9–12]. These suggest that co-administration concurrent with, or soon after, an inflammatory or septic insult can improve both morbidity and mortality. Ghrelin increased acute food intake in a rat model of thermal injury [9] with normalization of muscle atrogene expression and reductions in myofibrillar protein breakdown and pro-inflammatory cytokine levels [13]. This suggests that ghrelin may be able to limit muscle catabolism, a finding supported by limited studies in humans with chronic cachectic illnesses [14–18].

We thus sought to assess the impact of ghrelin treatment in a validated long-term rodent model of zymosan peritonitis [19]. This model recapitulates many aspects of human critical illness, including weight loss, muscle weakness and anorexia, which improves with clinical recovery [19]. We chose not to intervene in the immediate post-insult period as early nutrition has been associated with poor outcomes [20]. The aim of the study was to investigate the effects of ghrelin administration during the recovery phase of critical illness on food intake; body mass and body composition; muscle mass, functionality and histology; and hormonal, biochemical and immune levels.

## Methods

### Animals

Male Wistar rats (Charles River, Margate, UK) were singly housed in plastic cages at least 72 hours prior to sepsis induction. Rats had *ad libitum* access to food and water at all times. Standard rat chow was given (Harlan Teklad, Madison, WI) containing 18% protein and 5% fat. Temperature was controlled between 19–23°C and humidity at 55% (±10%), as were 12-hourly light/dark cycles. Rats were randomly allocated to groups receiving the septic insult and treatment with either ghrelin or placebo (n-saline) (Randomisation and blinding in S1 File). All studies were performed with approval from the local (University College London) Ethics Committee and the Home Office (UK) under the 1986 Scientific Procedures Act. Using the main outcome measure as increase in food intake, in order to detect a difference in food intake of 1 gram (SD 0.85g) with an alpha of 0.05 and a power of 90%, nine animals were required per group.

Critical illness was induced on Day 0 using zymosan (Sigma Aldrich, St. Louis, MO) (30 mg/100g body mass) mixed with liquid paraffin to a concentration of 25 mg/ml, and injected intraperitoneally via a 19G needle through the anterior abdominal wall, as previously described [19]. All injections were given under brief anesthesia with inhaled isoflurane. No antibiotic nor fluid resuscitation were given during the course of these experiments as this was a non-lethal model intended to focus upon the recovery phase.

Methods and measurements were performed as previously described [19]. In brief, animals and their food intake were weighed daily, with food intake being assessed from the weight of chow left in the food holder and on the cage floor. Animals were checked a minimum of four times daily while exhibiting signs of sepsis and clinically scored to assess the severity of the insult (Table 1) on a daily basis. Any animal in distress (scoring two consecutive ‘4’s or a ‘5’ on the severity scoring system) or unable to move, right itself or respond appropriately to external stimuli was immediately culled. All efforts were made to minimise unnecessary suffering of the experimental animals. Details of the number of animals used in each experiment are included in the Supporting Information (Animals in S1 File).

**Table 1:** The scoring system used to assess the degree of illness induced by zymosan.

Characteristic	Scoring range
Hunched	0–1
Bloated	0–1
Conjunctival injection	0–1
Piloerection	0–1
Lack of movement	0–2
Lack of alertness	0–2

Scoring denotes absence (0), presence (1), or where appropriate, marked presence (2). If an animal scored 5 it was reviewed more frequently and a decision was made before the end of the day as to whether it should be culled. Animals scoring 6 or more were culled immediately.

### Ghrelin delivery

Administration of human ghrelin (Bachem, Saffron Walden, Essex, UK) or placebo (n-saline) was commenced 48 hours after sepsis induction, by which time the majority of animals were showing early clinical signs of recovery e.g. increased appetite and interest in their surroundings. Ghrelin was diluted with saline to the appropriate concentration, based on effective doses used previously to increase food intake and body mass in rodent studies of health [21] and cachexia models of heart failure [22] and renal failure [23]. Subcutaneous osmotic mini-pumps (Alzet Models 2ML2 and 2002; Durect Co., Cupertino, CA) were used to administer the ghrelin continuously, with 100 nmol ghrelin (~300 nmol/kg) being delivered per 24 hours. Pumps were prepared as per the manufacturer’s instructions with saline-filled delay catheters attached to enable ghrelin release into the animal’s subcutaneous tissue to commence at 48 hours following implantation. The delay catheter consisted of polyethylene tubing, 0.76 mm internal diameter x 1.22 mm outside diameter (Linton Instrumentation, Diss, Norfolk, UK) cut to length to enable the 48-hour delay in administration. These pumps were implanted subcutaneously just prior to zymosan administration through a small skin incision made between the shoulder blades. A pocket to hold the pump was created by blunt dissection of the subcutaneous fascia. The incision was then closed with two 2–0 silk sutures. For analgesia, 0.2 ml of 0.5% xylocaine was injected locally.

### Ghrelin bioactivity

To confirm that ghrelin within the pumps remained bioactive during the course of the experiment, a separate bio-assay study was performed using fifteen additional male Wistar rats. On three consecutive days at the start of the light phase (07.00 am) rats were subcutaneously administered either saline (0.3 ml, vehicle, n = 5), freshly prepared ghrelin (100 nmol/0.3 ml, fresh, n = 5) or ghrelin removed from the osmotic mini-pumps of animals culled the day previously (100 nmol/0.3 ml, pump, n = 5). Food intake was recorded for one hour. The study was a cross-over design with animals rotated such that they each received each of the treatments on different days with a minimum 48-hour washout period between experiments.

### Measurement of muscle function, hormone levels and plasma biochemistry

Grip strength was measured using a grip strength meter (Linton, Diss, Norfolk, UK) pre-zymosan, and on Days 2, 5, 8 and 12 post-zymosan (Grip strength measurement in S1 File). Biochemical variables, ghrelin, insulin and leptin levels were measured in plasma on Day 12. Blood samples were taken after culling by decapitation (truncal blood: mixed arterial-venous) and prepared as previously described [19]. Plasma biochemistry was analysed by The Doctors Laboratory (London, UK) and the Department of Clinical Biochemistry, Charing Cross Hospital, London UK using standard analysers. Gut hormones, leptin and cytokines were measured in duplicate using rat-specific multiplex bead-based assays (Millipore, Billerica, MA). Data points greater than two standard deviations from the mean for each gut hormone and leptin at each timepoint were not included in the final analysis.

### Metabolic cart

In some studies, measurements were made of oxygen consumption (VO_2]_ and carbon dioxide production (VCO_2_) with subsequent calculation of the respiratory exchange ratio (VCO_2_ divided by VO_2_). Animals were placed in one of the four Comprehensive Laboratory Animal Monitoring System (CLAMS) metabolic carts (Columbus Instruments, Columbus, OH, USA). Naïve rats were placed in the metabolic carts for a two-hour period during the light phase on one occasion only. Rats receiving zymosan were placed in the metabolic cart for a two-hour period on Days 4, 7 and 12 (at the same time of day) during the light phase. The results for the first hour were excluded to allow for acclimatisation to the cage; the VO_2_ and VCO_2_ values of the second hour were averaged to give a mean VO_2_ and VCO_2_ for each animal on each day they were in the cart.

### Body composition & muscle histology

Total body composition was assessed at Day 12 in a sub-group of rats. Details of the body composition and histological analysis and are described in the Supporting Information (Body composition and Muscle histology in S1 File).

### Statistical analysis

Data were checked for normality using the Shapiro-Wilk test and presented as mean and standard error of the mean, or median and interquartile range, for parametric and non-parametric data, respectively. For comparisons between unpaired groups, Student’s t-tests and one- and two-way analysis of variance (ANOVA) was used with post hoc Bonferroni correction for parametric data, and the Mann-Whitney U and Kruskal-Wallis test with Dunn’s post hoc analysis for non-parametric data. Statistical analyses were performed with GraphPad Prism computer software (version 5.00 for Windows, GraphPad Software, San Diego, CA). Analysis of cumulative food intake and body mass was performed using the generalised estimating equation with Stata software (StataCorp. 2011. *Stata Statistical Software*: *Release 12*. College Station, TX, USA: StataCorp LP). Statistical significance was set at the 5% level.

## Results

### Outcomes and clinical severity

In total, 102 animals (body mass 264–333 g) were used (Animals in S1 File). The zymosan-vehicle and zymosan-ghrelin groups were equally affected in terms of clinical severity (mean severity score at 24 hours: 2.86±0.02 vs 2.63±0.27 respectively, p = 0.55).

### Bioactivity and delivery of ghrelin

**Fig 1:**
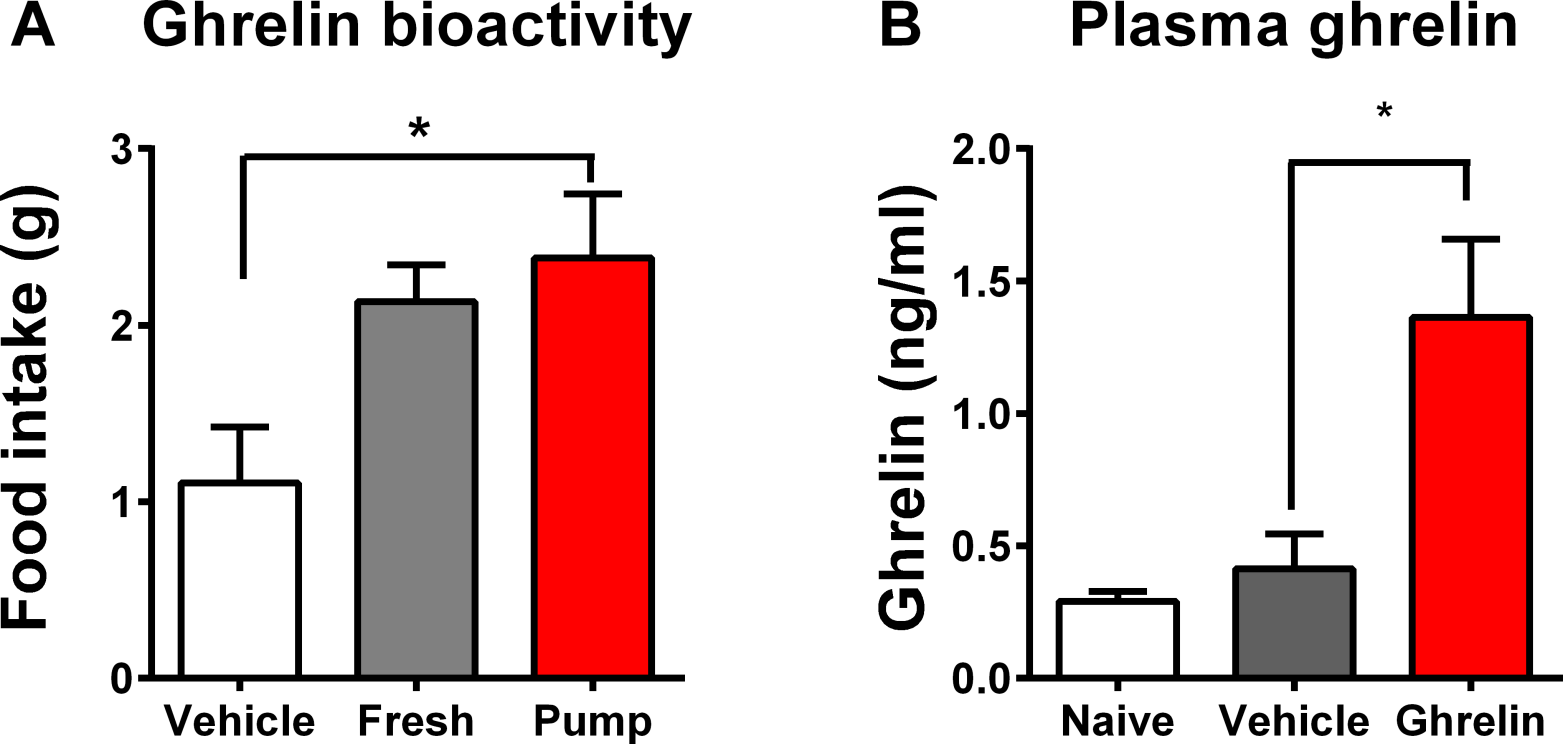
Ghrelin given via subcutaneous osmotic minipump induces acute food intake and enters the circulation. (A) Male Wistar rats (313–348 g) received a single SC injection of saline (0.3 ml, Vehicle, n = 5), freshly prepared ghrelin 100 nmol/0.3 ml, fresh, n = 5) or ghrelin recovered from the osmotic mini-pumps of animals culled after 12 days (100 nmol/0.3ml, Pump, n = 5). (B) Rats (274–333 g) received a single IP injection of zymosan (30 mg/100 g body mass) and were implanted with a subcutaneous mini-osmotic pump with a 48-hour delay catheter primed to infuse either saline (0.25 ul/ hour, zymosan-vehicle, n = 10) or ghrelin (0.25 ul/ hour; 100 nmol/day, zymosan-ghrelin, n = 12) on Day 0. Five animals had no intervention, shown for comparison (naïve). Data are expressed as mean (± SEM). P values refer to (A) one way ANOVA and (B) t-test between vehicle and ghrelin, * p<0.05.

One-hour food intake was higher (p = 0.012) in rats injected with pump-ghrelin (2.38 ±0.36 g) and fresh-ghrelin-treated animals (2.13 ±0.21 g) compared with vehicle controls (1.11 ±0.32 g) (Fig 1A), suggesting that ghrelin within the pump retained its biological activity over the course of study. Plasma ghrelin levels, measured at experiment end (Day 12), were significantly higher (p<0.05) in ghrelin-treated rats compared to saline controls (Fig 1B) indicating continued ghrelin delivery throughout the experimental period.

### Food intake and body mass

**Fig 2:**
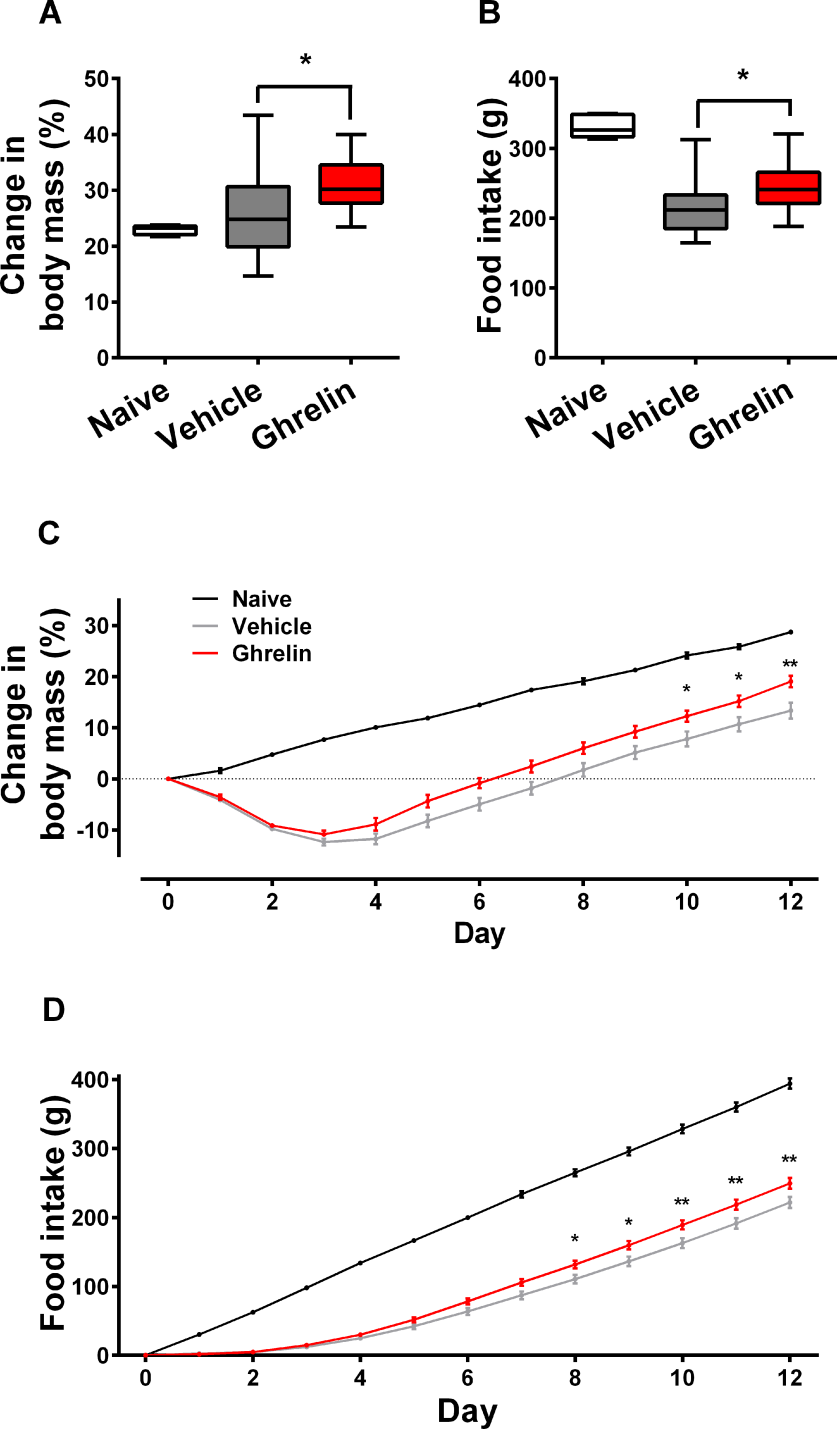
Effects on ghrelin on food intake and body mass following zymosan peritonitis. Male Wistar rats received either saline (zymosan-vehicle, n = 22) or ghrelin (zymosan-ghrelin, n = 19) after zymosan injection. Five animals had no intervention, shown for comparison (naïve). Total food intake and change in body mass (over days 2–12) are expressed as (A, B) median ± IQR (box) and range (whiskers). Cumulative food intake and change in body mass are shown as (C, D) mean ±SEM. P values refer to (A, B) t-test and (C, D) two-way ANOVA between vehicle and ghrelin, * p<0.05, ** p<0.01.

Between Days 2–12, when the ghrelin was being administered, there was a greater increase in body mass (Fig 2A) and food intake (Fig 2B) in zymosan-treated compared to zymosan-vehicle animals (p<0.05). Cumulative changes in body mass and food intake were significantly (p<0.0001) reduced in both zymosan groups compared to naïve and a significant trend was seen towards higher cumulative food intake and body mass in zymosan-treated animals given ghrelin compared to vehicle controls after Day 8 (Fig 2C and 2D). In addition, daily food intake corrected for body mass (between Days 2–12) and total food intake and weight gain (between Days 0–12) were significantly higher in the ghrelin-treated animals (S1 Fig).

### Muscle mass, body composition and muscle strength

**Fig 3:**
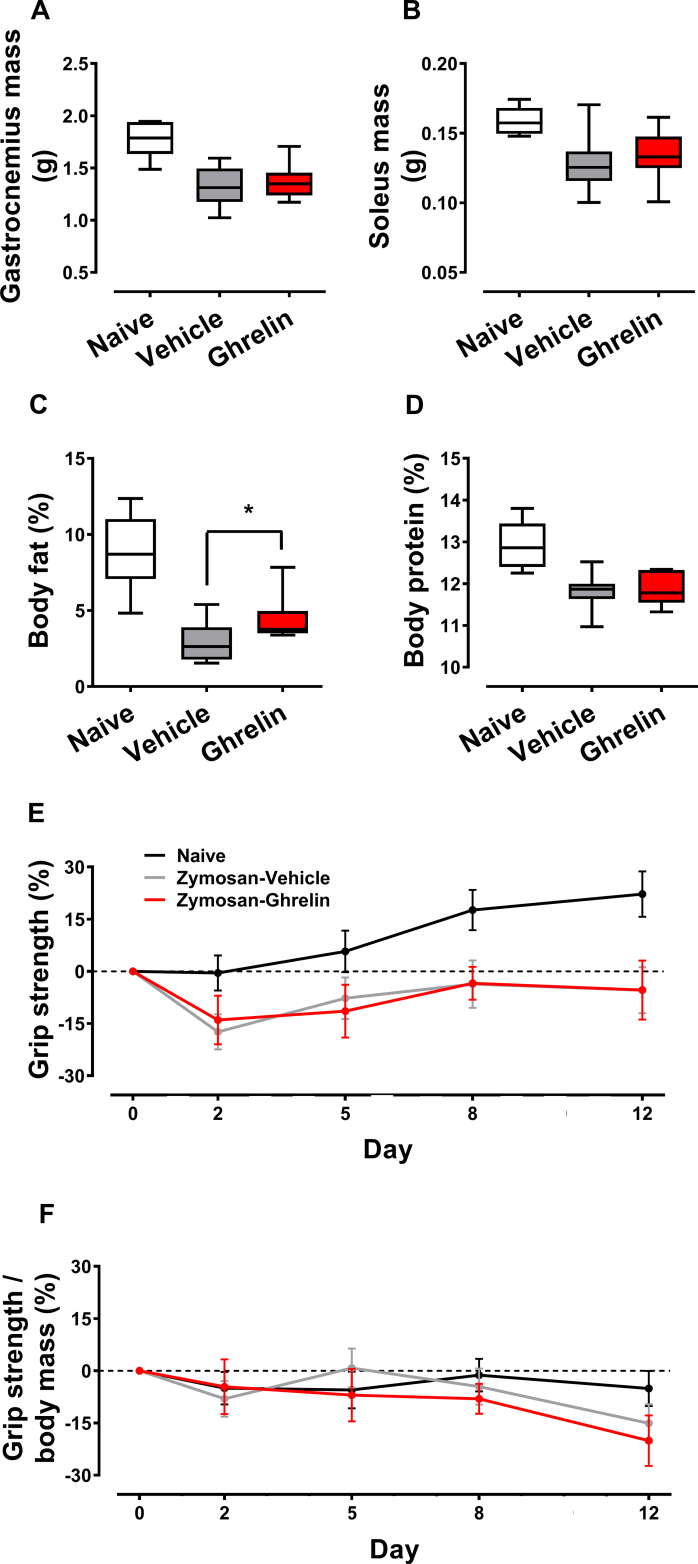
The effect of ghrelin treatment on body composition, muscle mass and forelimb grip strength following zymosan peritonitis. Male Wistar rats received either saline (zymosan-vehicle) or ghrelin (zymosan-ghrelin) after zymosan injection or no intervention (naïve) on Day 0. Data are expressed as mean (±SEM) for body composition and grip strength and as median ± IQR (box) and range (whiskers) for muscle mass. Wet mass of gastrocnemius (A) and soleus (B) (naïve n = 5, zymosan-vehicle n = 12, zymosan-ghrelin n = 7), percent body fat (C) and body protein (D) (naïve n = 8, zymosan-vehicle n = 10, zymosan-ghrelin n = 12) and grip strength (E) and grip strength adjusted for body mass (F) (naïve n = 5, zymosan-vehicle n = 9, zymosan-ghrelin n = 10) are shown. P value refers to t-test between vehicle and ghrelin, * p<0.05.

Zymosan-treated animals had a lower mass of gastrocnemius and soleus compared to naïve rats (Fig 3A and 3B). Ghrelin had no impact on the mass of individual leg muscles, even when correcting for body mass. The ratio of wet to dry muscle was similar between groups (data not shown), suggesting that changes in fluid content were not responsible for changes in muscle mass. In the sub-group used for body composition analysis, body fat and protein were lower in both ghrelin- and saline-treated zymosan rats compared to naïve rats (Fig 3C and 3D). While no difference was seen in body protein composition between ghrelin and saline-treated zymosan groups, the percentage of body fat was higher in those given ghrelin (p<0.05). The similar protein contents is in agreement with the lack of difference in muscle mass observed. Grip strength differed significantly between naïve and zymosan-treated rats (p<0.05); however, ghrelin had no impact on forelimb muscle strength (Fig 3E). There was no difference between groups when grip strength was corrected for body mass (Fig 3F).

### Histology

**Fig 4:**
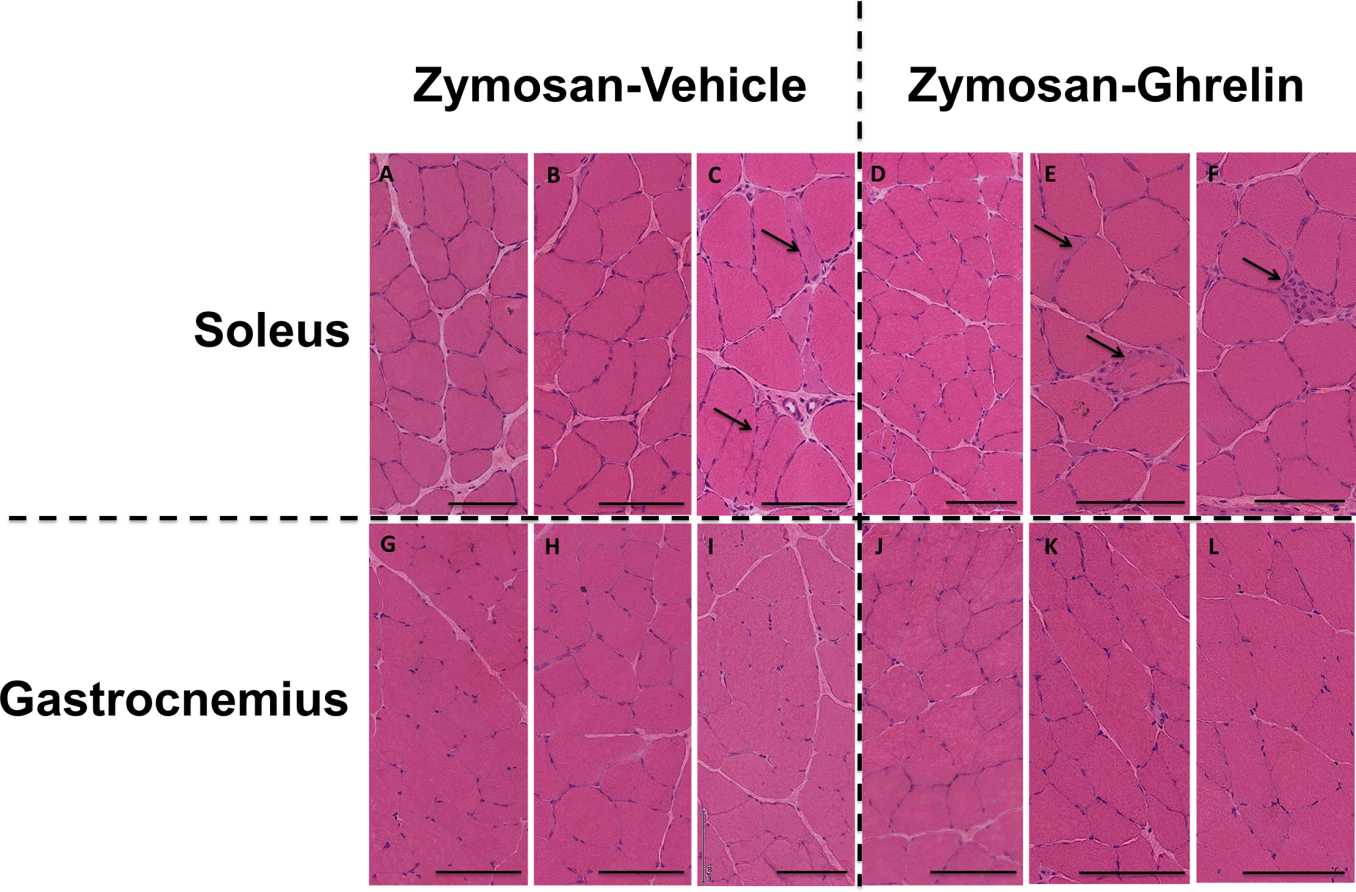
Examples of histopathological changes. The top row represent soleus (A-F) and bottom row shows the corresponding gastrocnemius (G-L). A-C and G-I were zymosan-vehicle treated animals; D-F and J-L were zymosan-ghrelin treated. A, B and D are normal, C shows mild pathology–rare atrophic fibers. E and F show rare atrophic fibers and also regenerating fibers. All gastrocnemius (G-L) in the bottom row are normal. The scale bar in each image equals 100 microns.

The histological appearances of the gastrocnemius and soleus muscle were unremarkable (Fig 4 and S1 and S2 Tables). No striking differences were seen between muscles of rats administered zymosan and then treated with saline, and those subsequently treated with ghrelin. In particular, gastrocnemius was histologically normal throughout. No specimen was obviously hypertrophic or had significant fiber size variation, and there was no florid inflammatory response. The fiber-typing pattern was similar throughout; soleus contains predominantly slow fibers and had a greater proportion of hybrid fibers. Within the gastrocnemius there was a consistently observed, well circumscribed area (close to the tendinous insertion) of mixed Type 1 and 2 fibers; the larger remaining area comprised (almost always) 100% fast fibers.

### Plasma biochemistry, cytokines, ghrelin, leptin, and insulin

Plasma cytokines, biochemistry, gut hormone and leptin levels were measured after culling the animals on Day 12 (S3 Table). No significant differences were seen in IL-6, IL-10 and INF-gamma (measured at Day 12) between any of the groups. Sodium was significantly higher in ghrelin treated-animals compared to animals receiving saline, whereas aspartate transaminase was lower in the ghrelin group (p<0.05). There were no differences in potassium, urea, creatinine, bicarbonate, alkaline phosphatase, alanine transaminase, triglycerides, cholesterol, HDL cholesterol, leptin or insulin. Ghrelin levels were higher in animals receiving ghrelin treatment (p<0.05).

### Metabolic monitoring

**Fig 5:**
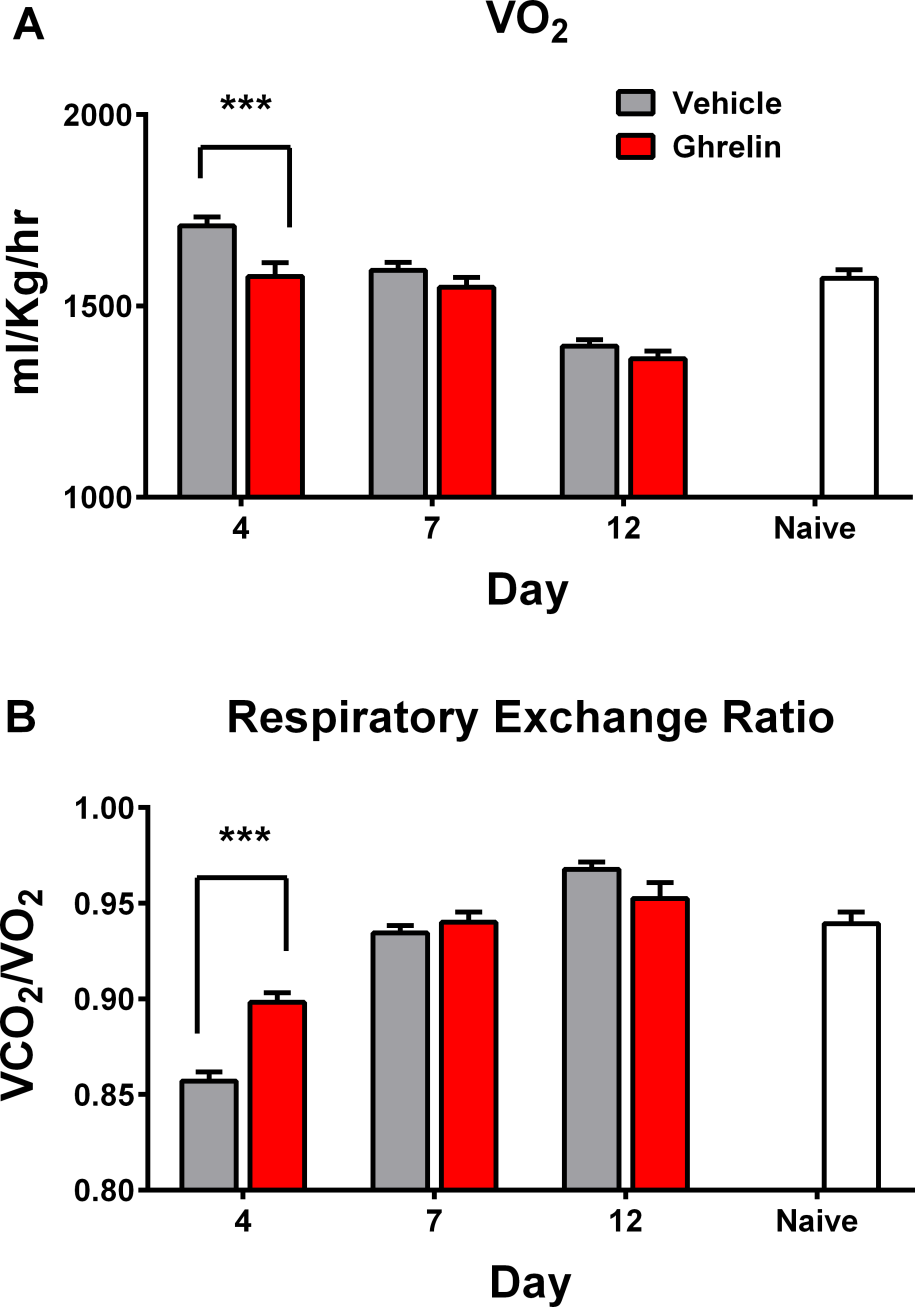
The effect of ghrelin treatment on metabolic parameters in animals with zymosan-induced peritonitis. Male Wistar rats received either saline (zymosan-vehicle) or ghrelin (zymosan-ghrelin) after zymosan injection. VO2 (A) and VCO2 were measured for one hour on Days 4, 7 and 12, and RER was calculated (B). Data are expressed as mean (±SEM). Eight animals had no intervention, shown for comparison (naïve). P value refers to t-test between vehicle and ghrelin, *** p<0.0001.

On Day 4 VO_2_ was significantly lower (and closer to that of naïve rats) in ghrelin-treated animals while the respiratory exchange ratio (RER) was significantly higher (p<0.0001) (Fig 5). However, no difference was seen on Days 7 and 12.

## Discussion

Administration of ghrelin during the recovery period increased food intake and body weight gain, and ameliorated the early effects on substrate metabolism in this rodent model of critical illness cachexia. However, ghrelin did not improve lean mass or muscle strength. We selected a dose of ghrelin that would achieve supraphysiologic levels and, importantly, chose not to start administering the agent too early as our *a priori* intention was to investigate rehabilitation in the post-critical illness phase, rather than treatment of the critical illness itself. This would increase relevance to the likely design of any future potential trial in critically ill patients. Selecting 48 hours as the time-point to commence ghrelin treatment after zymosan injection was based upon recovery of food intake and improvement in clinical scores from prior experiments. We chose to study the animals for 12 days based on timepoints utilized in our characterization study [19] and in pilot studies, however in human subject critical illness associated weakness can take months to recover from and it is possible that a longer period of study may yield different results. We felt that zymosan provided a preferable model of critical illness compared to the sepsis models that we piloted which tended to result in either death or rapid recovery. It is worth noting that, unlike our model which used healthy adolescent rats, human critical illness is multifactorial and often occurs in older people with established chronic diseases.

Others have previously reported that ghrelin increased daily food intake and cumulative body mass over 12 days [24]. In keeping with previous published work [25], we found ghrelin treatment did increase daily food intake compared to vehicle in animals recovering from critical illness, and this translated into significant gains in body mass during the treatment period. In a guinea pig model of critical illness administering higher levels of enteral feed at 48 hours after sepsis induction significantly increased mortality, even though more weight loss occurred in those animals fed lower energy diets [26]. We did not observe this in our study; the late (>48 hour) mortality of zymosan-animals treated with ghrelin or vehicle was similar.

Ghrelin use has increased weight gain and adiposity in other rodent models [27–29], including those with endotoxin-induced wasting [11]. The lack of a clear effect of ghrelin on either muscle mass or function in our study may relate in part to the decreased motor activity of the animals. Exercise enhances muscle protein synthesis following protein ingestion. It may be that the stimulus of exercise is required to potentiate ghrelin’s effects [30]. Another potential confounder is our use of a continuous subcutaneous infusion. In rat models of chronic renal failure, cancer cachexia and heart failure, subcutaneous daily injection of ghrelin over 5–28 day periods improved food intake, and total and lean body mass, usually without affecting fat mass [22,23,27–29] In their heart failure model, Akashi et al [22] found a continuous subcutaneous ghrelin infusion of 50 nmol/kg/day did not increase total body mass nor lean mass, but did increase fat mass, whereas 50 nmol/kg/day s.c. given three times daily increased body, lean and fat mass. However, in elderly (non-cachectic) patients, twice daily ghrelin administration for 1 week before and 2 weeks after total hip replacement failed to improve muscle strength, thigh muscle size, or walking ability, although lean body mass was increased and fat mass decreased compared to controls [31].

The body composition analysis showed that the additional body mass gained by the ghrelin-treated animals represents increased body fat rather than muscle. Whether changes in gastrocnemius and soleus muscle mass accurately reflects whole muscle mass is open to debate. However, the near-identical total protein content in ghrelin- and vehicle-treated rats supports their representativeness. Continuous intravenous infusion of ghrelin may promote increased adiposity (more than pulsatile delivery) [32] through attenuated increases in pulsatile growth hormone (GH) secretion known to occur with continuous ghrelin administration and a consequent decrease in GH-mediated lipolysis [32–33]. In the studies described above the effects of continuous subcutaneous ghrelin on receptor signalling are unknown. Persistent supraphysiological ghrelin levels may down-regulate its principal receptor (Growth Hormone Secretagogue Receptor-1a, GHSR-1a) and/or other receptors involved in feeding. If pulsatile GH secretion was down-regulated, this could affect the way that ghrelin partitions the additional energy gained by increased food intake, from lean to fat mass. Thus there is conflicting evidence as to whether ghrelin can increase lean body mass; dose, route, and frequency of administration appear important determinants.

The equivalent decrements in forelimb grip strength between ghrelin- and vehicle-treated rats indicate no functional gain associated with ghrelin treatment. This is unsurprising considering the failure to gain additional muscle mass in the ghrelin-treated animals. Even with ghrelin treatment, some animals had ongoing histological evidence of tissue damage in the soleus muscle after 12 days, further reflecting the significant challenges associated with rehabilitation in the post-critical illness phase when other clinical features and observations suggest recovery. Maurissen et al [34] previously showed that 24 days’ dietary restriction led to a significant reduction in forelimb grip strength, but this difference was lost when corrected for body mass. Our results, in which grip-strength difference between zymosan-treated and naïve animals is maintained even when corrected for body mass, suggest that weight loss alone is unlikely to have accounted for the reduction in grip strength observed in the zymosan-treated rats. The effects of zymosan-induced inflammation, feeling unwell and other psychological effects may have contributed.

Ghrelin did not influence overall oxygen consumption in our model although it did reduce oxygen consumption and increase RER at the Day 4 timepoint. Accepting the caveats of using RER as a marker of the respiratory quotient, this result is biologically plausible, i.e. ghrelin-stimulated increases in food intake would provide additional carbohydrate for fuel utilization which might increase the RER, and that ghrelin may independently promote switching substrate metabolism from fat to carbohydrate. This may in part explain why the increased food intake resulted in fat gain, although it is not possible to discern this because of the changes in food intake. Alternative explanations for these findings include activation of enzymes associated with fat storage, or lowering of thermogenesis and energy expenditure through reduced expression of uncoupling proteins in brown adipose tissue [35–36]. Ghrelin may also inhibit lipolysis through inhibition of sympathetic nervous activity [37]. We were not able to collect data on nitrogen balance, which would have provided useful additional information.

In summary, although exogenous ghrelin commenced two days after a septic insult was able to stimulate food intake and significantly increase body mass, this additional weight gain was principally due to an increase in body fat with no improvements in muscle mass or muscle strength. Further studies are required to determine whether pulsatile ghrelin in combination with, for example, exercise, may have beneficial anabolic effects in critical illness.

## Supporting information

S1 File Supporting information. (DOCX)

S1 Table Morphological appearance of soleus and gastrocnemius muscle after H&E staining in Zymosan-Vehicle treated rats. Muscle was frozen in melting 2-methylbutarate and later sectioned (8 micron thickness) then stained with H&E.
(DOCX)

S2 Table Morphological appearance of soleus and gastrocnemius muscle after H&E staining in Zymosan-Ghrelin treated rats. Muscle was frozen in melting 2-methylbutarate and later sectioned (8 micron thickness) then stained with H&E.
(DOCX)

S3 Table Effect of ghrelin treatment on plasma biochemistry, gastrointestinal hormones and leptin after a zymosan peritonitis. Male Wistar rats (274–333 g) received a single injection of either zymosan (30 mg/100 g body mass) or saline, and were implanted with a subcutaneous mini-osmotic pump with a 48-hour delay catheter primed to infuse either saline (0.25 ul/ hour, Zymosan-Vehicle, n = 10) or ghrelin (100 nmol/day, Zymosan-Ghrelin, n = 12) on Day 0. Five animals had no intervention, shown for comparison (Naïve). Animals were culled on Day 12. Data are expressed as mean (±SEM). P value refers to t-test between vehicle and ghrelin, * p<0.05.
(DOCX)

S1 Fig Effects on ghrelin on food intake and body mass following zymosan peritonitis. Male Wistar rats (264–333 g) received a single injection of zymosan (30 mg/100 g b.w.) and were implanted with a SC mini-osmotic pump with a 48-hour delay catheter primed to infuse either saline (0.25 ul/ hour, zymosan-vehicle, n = 22) or ghrelin (0.25 ul/ hour; 100 nmol/day, zymosan-ghrelin, n = 19) on Day 0. Five animals had no intervention, shown for comparison (naïve). Animals were culled on Day 12. Daily food intake corrected for body mass between Days 2–12 (A), and total food intake (B) and total weight gain (C) between Days 0–12 are shown. Data are expressed as median ± IQR (box) and range (whiskers). P values refer to t-test between vehicle and ghrelin, * p<0.05, ** p<0.01.
(TIF)

## References

[1] Herridge MS, Cheung AM, Tansey CM, Matte-Martyn A, Diaz-Granados N, Al-Saidi F, et al One-year outcomes in survivors of the acute respiratory distress syndrome. New Engl J Med. 2003;348: 683–693. doi: 10.1056/NEJMoa022450 12594312 10.1056/NEJMoa022450

[2] Kress JP, Hall JB. ICU-acquired weakness and recovery from critical illness. N Engl J Med. 2014;370: 1626–1635. doi: 10.1056/NEJMra1209390 24758618 10.1056/NEJMra1209390

[3] Herndon DN, Hart DW, Wolf SE, Chinkes DL, Wolfe RR. Reversal of catabolism by beta-blockade after severe burns. N Engl J Med. 2001;345: 1223–1229. doi: 10.1056/NEJMoa010342 11680441 10.1056/NEJMoa010342

[4] Przkora R, Jeschke MG, Barrow RE, Suman OE, Meyer WJ, Finnerty CC, et al Metabolic and hormonal changes of severely burned children receiving long-term oxandrolone treatment. Ann Surg. 2005;242: 384–389. doi: 10.1097/01.sla.0000180398.70103.24 16135924 10.1097/01.sla.0000180398.70103.24 PMC1357746

[5] Takala J, Ruokonen E, Webster NR, Nielsen MS, Zandstra DF, Vundelinckx G, et al Increased mortality associated with growth hormone treatment in critically ill adults. New Engl J Med. 1999;341: 785–792. doi: 10.1056/NEJM199909093411102 10477776 10.1056/NEJM199909093411102

[6] Acker CG, Singh AR, Flick RP, Bernardini J, Greenberg A, Johnson JP. A trial of thyroxine in acute renal failure. Kidney Int. 2000;57: 293–298. doi: 10.1046/j.1523-1755.2000.00827.x 10620211 10.1046/j.1523-1755.2000.00827.x

[7] Casaer MP, Van den Berghe G. Nutrition in the acute phase of critical illness. N Engl J Med. 2014;370, 1227–1236. doi: 10.1056/NEJMra1304623 24670169 10.1056/NEJMra1304623

[8] Nematy M, O'Flynn JE, Wandrag L, Brynes AE, Brett SJ, Patterson M, et al Changes in appetite related gut hormones in intensive care unit patients: a pilot cohort study. Crit Care. 2005;10: R10. 10.1186/cc3957 PMC1550795 16420657

[9] Balasubramaniam A, Wood S, Joshi R, Su C, Friend LA, Sheriff S, et al Ghrelin stimulates food intake and growth hormone release in rats with thermal injury: synthesis of ghrelin. Peptides. 2006;27: 1624–1631. doi: 10.1016/j.peptides.2006.02.005 16574277 10.1016/j.peptides.2006.02.005

[10] Chang L, Du JB, Gao LR, Pang YZ, Tang CS. Effect of ghrelin on septic shock in rats. Acta Pharmacol Sin. 2003;24: 45–49. 12511228

[11] Chorny A, Anderson P, Gonzalez-Rey E, Delgado M. Ghrelin protects against experimental sepsis by inhibiting high-mobility group box 1 release and by killing bacteria. J Immunol. 2008;180: 8369–8377. 18523304 10.4049/jimmunol.180.12.8369

[12] Hataya Y, Akamizu T, Hosoda H, Kanamoto N, Moriyama K, Kangawa K, et al Alterations of plasma ghrelin levels in rats with lipopolysaccharide-induced wasting syndrome and effects of ghrelin treatment on the syndrome. Endocrinol. 2003;144: 5365–5371. 10.1210/en.2003-0427 12960078

[13] Balasubramaniam A, Joshi R, Su C, Friend LA, Sheriff S, Kagan RJ, et al Ghrelin inhibits skeletal muscle protein breakdown in rats with thermal injury through normalizing elevated expression of E3 ubiquitin ligases MuRF1 and MAFbx. Am J Physiol Regul Integr Comp Physiol. 2009;296: R893–R901. doi: 10.1152/ajpregu.00015.2008 19211729 10.1152/ajpregu.00015.2008

[14] Ashby DR, Ford HE, Wynne KJ, Wren AM, Murphy KG, Busbridge M, et al Sustained appetite improvement in malnourished dialysis patients by daily ghrelin treatment. Kidney Int. 2009;76: 199–206. doi: 10.1038/ki.2009.114 19387475 10.1038/ki.2009.114

[15] Nagaya N, Itoh T, Murakami S, Oya H, Uematsu M, Miyatake K, et al Treatment of Cachexia With Ghrelin in Patients With COPD. Chest. 2005;128: 1187–1193. doi: 10.1378/chest.128.3.1187 16162705 10.1378/chest.128.3.1187

[16] Neary NM, Small CJ, Wren AM, Lee JL, Druce MR, Palmieri C, et al Ghrelin increases energy intake in cancer patients with impaired appetite: acute, randomised, placebo-controlled trial. J Clin Endocrinol Metab. 2004;89: 2832–2836. doi: 10.1210/jc.2003-031768 15181065 10.1210/jc.2003-031768

[17] Miki K, Maekura R, Nagaya N, Nakazato M, Kimura H, Murakami S, et al Ghrelin treatment of cachectic patients with chronic obstructive pulmonary disease: A multicenter, randomised, double-blind, placebo-controlled trial. PloS One. 2012;7: e35708 doi: 10.1371/journal.pone.0035708 22563468 10.1371/journal.pone.0035708 PMC3341383

[18] Wynne K, Giannitsopoulou K, Small CJ, Patterson M, Frost G, Ghatei MA, et al Subcutaneous ghrelin enhances acute food intake in malnourished patients who receive maintenance peritoneal dialysis: a randomised, placebo-controlled trial. J Am Soc Nephrol. 2005;16: 2111–2118. doi: 10.1681/ASN.2005010039 15888560 10.1681/ASN.2005010039

[19] Hill NE, Saeed S, Phadke R, Ellis MJ, Chambers D, Wilson DR, et al Detailed characterization of a long-term rodent model of critical illness and recovery. Crit Care Med. 2015;43: e84–96. doi: 10.1097/CCM.0000000000000854 25700075 10.1097/CCM.0000000000000854

[20] Casaer MP, Mesotten D, Hermans G, Wouters PJ, Schetz M, Meyfroidt G, et al Early versus late parenteral nutrition in critically ill adults. New Engl J Med. 2011;365: 506–517. doi: 10.1056/NEJMoa1102662 21714640 10.1056/NEJMoa1102662

[21] Strassburg S, Anker SD, Castaneda TR, Burget L, Perez-Tilve D, Pfluger PT, et al Long-term effects of ghrelin and ghrelin receptor agonists on energy balance in rats. Am J Physiol. 2008;295: E78–E84. 10.1152/ajpendo.00040.2008 PMC2493589 18460598

[22] Akashi YJ, Palus S, Datta R, Halem H, Taylor JE, Thoene-Reineke C, et al No effects of human ghrelin on cardiac function despite profound effects on body composition in a rat model of heart failure. Int J Cardiol. 2009;137: 267–275. doi: 10.1016/j.ijcard.2008.06.094 18723230 10.1016/j.ijcard.2008.06.094

[23] DeBoer MD, Zhu X, Levasseur PR, Inui A, Hu Z, Han G, et al Ghrelin treatment of chronic kidney disease: improvements in lean body mass and cytokine profile. Endocrinol. 2008;149: 827–35. 10.1210/en.2007-1046 PMC2219314 18039782

[24] Nakazato M, Murakami N, Date Y, Kojima M, Matsuo H, Kangawa K, et al A role for ghrelin in the central regulation of feeding. Nature. 2001;409: 194–198. doi: 10.1038/35051587 11196643 10.1038/35051587

[25] Wren AM, Small CJ, Abbott CR, Dhillo WS, Seal LJ, Cohen MA, et al Ghrelin causes hyperphagia and obesity in rats. Diabetes. 2001;50: 2540–2547. 11679432 10.2337/diabetes.50.11.2540

[26] Alexander JW, Gonce SJ, Miskell PW, Peck MD, Sax H. A new model for studying nutrition in peritonitis. The adverse effect of overfeeding. Ann Surg. 1989;209: 334–340. 2493777 10.1097/00000658-198903000-00014 PMC1493937

[27] DeBoer MD, Zhu XX, Levasseur P, Meguid MM, Suzuki S, Inui A, et al Ghrelin treatment causes increased food intake and retention of lean body mass in a rat model of cancer cachexia. Endocrinol. 2007;148: 3004–3012. 10.1210/en.2007-0016 17347304

[28] Barazzoni R, Zhu XX, DeBoer M, Datta R, Culler MD, Zanetti M, et al Combined effects of ghrelin and higher food intake enhance skeletal muscle mitochondrial oxidative capacity and AKT phosphorylation in rats with chronic kidney disease. Kidney Int. 2010;77: 23–28. doi: 10.1038/ki.2009.411 19890275 10.1038/ki.2009.411 PMC2857601

[29] Nagaya N, Uematsu M, Kojima M, Date Y, Nakazato M, Okumura H, et al Elevated circulating level of ghrelin in cachexia associated with chronic heart failure: relationships between ghrelin and anabolic/catabolic factors. Circulation. 2001;104: 2034–2038. 11673342 10.1161/hc4201.097836

[30] Witard OC, Jackman SR, L Breen, Smith K, Selby A, Tipton KD. Myofibrillar muscle protein synthesis rates subsequent to a meal in response to increasing doses of whey protein at rest and after resistance exercise. Am J Clin Nutr. 2014;99: 86–95. doi: 10.3945/ajcn.112.055517 24257722 10.3945/ajcn.112.055517

[31] Akamizu T, Iwakura H, Ariyasu H, Murayama T, Sumi E, Teramukai S, et al Effects of ghrelin treatment on patients undergoing total hip replacement for osteoarthritis: different outcomes from studies in patients with cardiac and pulmonary cachexia. J Am Geriatr Soc. 2008;56: 2363–2365. doi: 10.1111/j.1532-5415.2008.02031.x 19093947 10.1111/j.1532-5415.2008.02031.x

[32] Thompson NM, Gill DAS, Davies R, Loveridge N, Houston PA, Robinson IC, et al Ghrelin and des-octanoyl ghrelin promote adipogenesis directly in vivo by a mechanism independent of the type 1a growth hormone secretagogue receptor. Endocrinol. 2004;145: 234–242. 10.1210/en.2003-0899 14551228

[33] Thompson NM, Davies JS, Mode A, Houston PA, Wells T. Pattern-dependent suppression of growth hormone (GH) pulsatility by ghrelin and GH-releasing peptide-6 in moderately GH-deficient rats. Endocrinol. 2003;44: 4859–4867. 10.1210/en.2003-0423 12960077

[34] Maurissen JPJ, Marable BR, Andrus AK, Stebbins KE. Factors affecting grip strength testing. Neurotoxicol Teratol. 2003;25: 543–553. 12972067 10.1016/s0892-0362(03)00073-4

[35] Frayn KN. Hormonal control of metabolism in trauma and sepsis. Clin Endocrinol. 1986;24: 577–599. 10.1111/j.1365-2265.1986.tb03288.x 3539414

[36] Theander-Carrillo C, Wiedmer P, Cettour-Rose P, Nogueiras R, Perez-Tilve D, Pfluger P, et al Ghrelin action in the brain controls adipocyte metabolism. J Clin Invest. 2006;116: 1983–1993. doi: 10.1172/JCI25811 16767221 10.1172/JCI25811 PMC1474815

[37] Wu R, Zhou M, Das P, Dong W, Ji Y, Yang D, et al Ghrelin inhibits sympathetic nervous activity in sepsis. Am J Physiol. 2007;293: E1697–1702. 10.1152/ajpendo.00098.2007 17911350

