# Supplementary material for: Impact of ghrelin on body composition and muscle function in a long-term rodent model of critical illness
Source: PLoS One. 2017 Aug 10;12(8):e0182659. doi: 10.1371/journal.pone.0182659 (PMC5552127; doi:10.1371/journal.pone.0182659)
Supplement: S1 File — (DOCX) [file pone.0182659.s001.docx]

**Supporting Information**

**Animals**

In total 102 animals were used. Fifteen were used exclusively for the assay to assess ghrelin bioactivity. Eight were used as naive controls solely for the metabolic cart study. Of the remaining 79 animals, on Day 0, 74 animals were randomly allocated to receive IP zymosan/liquid paraffin mix (zymosan) or vehicle injected under isoflurane anesthesia. These animals were then randomized to receive a mini-osmotic pump with a 48-hour delay catheter primed to infuse subcutaneously either saline (0.25 ±l/ hour, zymosan-vehicle, n=35) or ghrelin (0.25 ±l/ hour; 100 nmol/day, zymosan-ghrelin, n=39). The imbalance in numbers was due to the unpredictability of animal deaths between the two groups. In the zymosan-vehicle group, five of 35 animals died, while eight were excluded (as they had a clinical severity score of 0 at 24 hours). In the zymosan-ghrelin group, 14 of the 39 animals died, four were excluded because of a 24 hour severity score of 0, while two were excluded due to recurrence of illness and weight loss after initial recovery. Four animals were culled due to illness severity (n=1 zymosan-vehicle; n=3 zymosan-ghrelin). The higher death rate in the zymosan-ghrelin group was not statistically different (p>0.05, Fishers exact test) from the zymosan-vehicle group and was felt extremely unlikely to be due to ghrelin treatment itself, as 12 out of the 14 deaths occurred within the first 48 hours; during this time the delay catheter on the mini-pump was delivering saline to both groups. Five animals had no intervention (naïve, n=5).

**Randomisation and blinding**

Once singly housed, animals were allocated a specific number before the experiments started. They were randomised by selecting numbers printed on folded paper from a deep container, to join each experimental group. This allocation was used during the day of minipump insertion to ensure the animals received the appropriate treatment. Thereafter, the researcher did not have access to the allocations when undertaking further measurements. Due to the relatively large number of animals being used during each wave of experiments, the researcher could not recall which animals had received the minipumps containing ghrelin and which contained the saline vehicle, thus was partly blinded from the allocation of each animal. It was not possible to blind the study as one researcher carried out almost all of the experimental procedures and measurements. The histopathologist (RP) was fully blinded.

**Grip strength measurement**

Muscle function tests were performed using a grip strength meter (Linton Instrumentation, Diss, Norfolk, UK) on Days 2, 5, 8 and 12. Familiarization with the grip strength meter was ensured over a week prior to study commencement. The forelimbs were placed on a T-bar attached to a force transducer shaft connected to a peak amplifier, and allowed to flex before being pulled horizontally away gently by the base of the tail [34]. The maximum grip force exerted by the rat until it released its grip was recorded. This was repeated five times for each animal (at 20 second intervals). The mean maximum peak force (in grams) was calculated from the top three values obtained [34].

**Body composition**

Rats were culled by cervical dislocation under isoflurane anesthesia. The contents of the gastrointestinal tract were cleared manually, and any intra-abdominal fluid removed. The carcass was dissolved in 1 ml 3M KOH (Sigma) in 65% ethanol (VWR, Radnor, Pennsylvania, PA) per 1 g carcass mass, sealed in a plastic pot and placed in an oven at 70°C for 5 days. Bones were removed by passing the liquid through a sieve and the total amount of liquid was then made up to 1000 ml by addition of 100% ethanol. From this a sample of fluid was removed and stored for analysis of protein and fat content. Carcass protein was determined using a modified Lowry Protein Assay kit (Thermo Scientific, Rockford, IL). The microplate containing the samples was read on a Synergy 2 plate reader (Biotek, Winooski, VT) at 630 nm. To determine carcass fat content a glycerol assay kit (Randox, Crumlin, Co. Antrim, UK) was used. The plate containing the samples was read at 520 nm on a plate reader (Labsystem Multiskan RC, Thermo Lab Systems, Franklin, MA).

**Muscle histology**

Histological analysis was undertaken in the zymosan-treated rats to examine the effects of ghrelin treatment on muscle fibre size and type compared to saline control. After culling, gastrocnemius and soleus muscles were rapidly dissected and weighed, before freezing in melting 2-methylbutarate (Merck) and then stored at -80°C. Later, the muscles were mounted on metal chucks embedded in optimal cutting temperature compound (Sakura Finetek, Thatcham, Berks, UK) and sectioned (6 and 8 micron thickness) on a cryostat (Model OTF, Bright, Huntingdon, Cambs, UK).

For histological examination, slides with 8 micron sections were stained using an automated stainer (IntelliPATH FLXTM, A. Menarini Diagnostics, Wokingham, Berks, UK) with hematoxylin and eosin as previously described [19]. Immunocytochemistry was performed on 6 micron sections to differentiate fast and slow myosin. Mouse monoclonal antibodies were used for both fast myosin (Novocastra NCL-MHCf, Leica Biosystems, Milton Keynes, Beds UK) diluted 1:50 in universal diluent (A. Menarini Diagnostics), and slow myosin (Novocastra NCL-MHCs, Leica Biosystems) diluted 1:20. Qualitative histopathological changes and morphological differences were assessed in a blinded fashion by a consultant histopathologist (RP).
