# Supplementary material for: Impact of ghrelin on body composition and muscle function in a long-term rodent model of critical illness
Source: PLoS One. 2017 Aug 10;12(8):e0182659. doi: 10.1371/journal.pone.0182659 (PMC5552127; doi:10.1371/journal.pone.0182659)
Supplement: S1 Table — Muscle was frozen in melting 2-methylbutarate and later sectioned (8 micron thickness) then stained with H&E. (DOCX) [file pone.0182659.s002.docx]

| **Vehicle** | | |
| --- | --- | --- |
| **No.** | **Soleus** | **Gastrocnemius** |
| V1 | Normal appearance | Normal appearance |
| V2 | Scattered inflammatory cells in peri-myseal septa with possible edema. Occasional smaller fibers | Normal appearance |
| V3 | Missing | Normal appearance |
| V4 | Normal appearance | Normal appearance |
| V5 | Normal appearance | Normal appearance |
| V6 | Normal appearance | Normal appearance |
| V7 | Normal appearance | Normal appearance |
| V8 | Single necrotic/regenerating fiber; otherwise normal | Normal appearance |
| V9 | More fiber size variation - frequent small fibers | Normal appearance |
| V10 | Normal appearance | Normal appearance |
