# Supplementary material for: Impact of ghrelin on body composition and muscle function in a long-term rodent model of critical illness
Source: PLoS One. 2017 Aug 10;12(8):e0182659. doi: 10.1371/journal.pone.0182659 (PMC5552127; doi:10.1371/journal.pone.0182659)
Supplement: S2 Table — Muscle was frozen in melting 2-methylbutarate and later sectioned (8 micron thickness) then stained with H&E. (DOCX) [file pone.0182659.s003.docx]

| **Ghrelin** | | |
| --- | --- | --- |
| **No.** | **Soleus** | **Gastrocnemius** |
| G1 | Some smaller fibers; scattered necrotic fibers | Normal appearance |
| G2 | One necrotic fiber, occasional lobulated type fibers | Normal appearance |
| G3 | One necrotic fiber, few peri-myseal inflammatory cells | Normal appearance |
| G4 | One hypercontracted fiber (pre-degeneration); possible lobulated type fibers; otherwise normal appearance | Normal appearance |
| G5 | Normal appearance | Normal appearance |
| G6 | Small group of 3-4 small fibers; rest normal appearance | Normal appearance |
| G7 | Normal appearance | Normal appearance |
| G8 | Normal appearance | Normal appearance |
| G9 | Normal appearance | Normal appearance |
| G10 | Normal appearance | Normal appearance |
| G11 | Normal appearance | Normal appearance |
| G12 | Normal appearance | Normal appearance |
