# Supplementary material for: Impact of ghrelin on body composition and muscle function in a long-term rodent model of critical illness
Source: PLoS One. 2017 Aug 10;12(8):e0182659. doi: 10.1371/journal.pone.0182659 (PMC5552127; doi:10.1371/journal.pone.0182659)
Supplement: S3 Table — Male Wistar rats (274–333 g) received a single injection of either zymosan (30 mg/100 g body mass) or saline, and were implanted with a subcutaneous mini-osmotic pump with a 48-hour delay catheter primed to infuse either saline (0.25 ul/ hour, Zymosan-Vehicle, n = 10) or ghrelin (100 nmol/day, Zymosan-Ghrelin, n = 12) on Day 0. Five animals had no intervention, shown for comparison (Naïve). Animals were culled on Day 12. Data are expressed as mean (±SEM). P value refers to t-test between vehicle and ghrelin, * p<0.05. (DOCX) [file pone.0182659.s004.docx]

|  | **Naïve** | **Vehicle** | **Ghrelin** |
| --- | --- | --- | --- |
| Sodium (mmol/l) | 140.4 (0.9) | 139.8 (0.6) | 141.4 * (0.4) |
| Potassium (mmol/l) | 6.8 (0.1) | 6.5 (0.2) | 6.3 (0.1) |
| Urea (mmol/l) | 6.3 (0.2) | 5.2 (0.2) | 5.0 (0.2) |
| Creatinine (umol/l) | 37.8 (0.5) | 36.1 (0.4) | 35.8 (0.4) |
| Bicarbonate (mmol/l) | 25.2 (0.7) | 25.0 (0.4) | 25.7 (0.4) |
| ALP (IU/L) | 230.6 (16.3) | 190.4 (7.2) | 201.0 (10.1) |
| ALT (IU/L) | 57.8 (2.5) | 47.1 (2.1) | 39.8 (3.1) |
| AST (IU/L) | 142.8 (16.6) | 132.6 (10.3) | 109.1 * (3.3) |
| Triglycerides (mmol/l) | 1.34 (0.24) | 1.54 (0.13) | 1.96 (0.22) |
| Cholesterol (mmol/l) | 2.43 (0.21) | 2.26 (0.09) | 2.29 (0.09) |
| HDL cholesterol (mmol/l) | 0.76 (0.04) | 0.63 (0.03) | 0.61 (0.03) |
| Ghrelin (ng/ml) | 0.29 (0.04) | 0.41 (0.13) | 1.36 * (0.30) |
| Leptin (ng/ml) | 9.66 (1.39) | 1.88 (0.28) | 2.63 (0.31) |
| Insulin (ng/ml) | 3.17(0.35) | 1.65 (0.16) | 1.73 (0.28) |
